# From food to pest: Conversion factors determine switches between ecosystem services and disservices

**DOI:** 10.1007/s13280-016-0813-6

**Published:** 2016-09-02

**Authors:** Laura Vang Rasmussen, Andreas E. Christensen, Finn Danielsen, Neil Dawson, Adrian Martin, Ole Mertz, Thomas Sikor, Sithong Thongmanivong, Pheang Xaydongvanh

**Affiliations:** 1 Department of Geosciences and Natural Resource Management, University of Copenhagen, Øster Voldgade 10, 1350 Copenhagen K, Denmark; 2 International Forestry Resources and Institutions (IFRI), School of Natural Resources & Environment, University of Michigan, 440 Church St., Ann Arbor, MI 48109 USA; 3 Nordic Agency for Development and Ecology (NORDECO), Skindergade 23, 1159 Copenhagen K, Denmark; 4 School of International Development, University of East Anglia, Norwich Research Park, Norwich, NR4 7TJ UK; 5 Faculty of Forestry, National University of Laos, Dongdok, Xaythany District, Vientiane, Laos

**Keywords:** Cash crop production, Conservation, Ecosystem disservices, Ecosystem services, Shifting cultivation

## Abstract

Ecosystem research focuses on goods and services, thereby ascribing beneficial values to the ecosystems. Depending on the context, however, outputs from ecosystems can be both positive and negative. We examined how provisioning services of wild animals and plants can switch between being services and disservices. We studied agricultural communities in Laos to illustrate when and why these switches take place. Government restrictions on land use combined with economic and cultural changes have created perceptions of rodents and plants as problem species in some communities. In other communities that are maintaining shifting cultivation practices, the very same taxa were perceived as beneficial. We propose conversion factors that in a given context can determine where an individual taxon is located along a spectrum from ecosystem service to disservice, when, and for whom. We argue that the omission of disservices in ecosystem service accounts may lead governments to direct investments at inappropriate targets.

**Electronic supplementary material:**

The online version of this article (doi:10.1007/s13280-016-0813-6) contains supplementary material, which is available to authorized users.

## Introduction

Research on connections between ecosystems and human wellbeing has focused on the beneficial goods and services provided by nature (MA 2005; Sachs and Reid 2006; Harrison et al. 2014). In this paper, we think of nature’s ecological functions as providing ‘outputs’ for humans. These ecosystem outputs can be demonstrably beneficial or harmful as ecosystems can also provide disservices (Lyytimäñki and Sipiläñ 2009; Cumming et al. 2014; Lyytimäñki 2014; Shapiro and Báldi 2014; Sandbrook and Burgess 2015). Disservices include, for example, crop pests and pathogens and weeds (Zhang et al. 2007; Dunn 2010). Failure to fully recognize disservices has potentially important consequences for governance of land and resources (Saunders et al. 2015) as harmful outputs or disservices may outweigh beneficial services for those living adjacent to forest ecosystems. Yet, there is limited empirical evidence available on ecosystems that at the same time provide both beneficial and harmful services to the same people (Villa et al. 2014)—although multiple programs such as the Community Areas Management Programme for Indigenous Resources (CAMPFIRE) (Child 1996) and an extensive body of scholarly work (e.g., Treves et al. 2006) have recognized and addressed the interlinked problems of e.g., wildlife crop damage and wildlife recreation. Instead, much attention has been given to how government agencies should manage ecosystems like forests and identify and respond to trade-offs defined as occurring where management of an area enhances one or more services at the cost of other services (Howe et al. 2014). Such efforts overlook the important dimension to ecosystem trade-offs, occurring between services and disservices (Ango et al. 2014).

To enhance understandings of the linkages between ecosystems and wellbeing, is it then enough just to acknowledge the presence of disservices? We believe it is important to note that although the terms ‘ecosystem services’ and ‘disservices’ imply that the services are a function of ecological processes, the positive or negative effects are in fact influenced by social as well as ecological processes. It has even been suggested that ecosystem services might be better termed ‘social–ecological services’ (Huntsinger and Oviedo 2014). Accordingly, recent studies have called for a broadening of ecosystem service frameworks by highlighting how social, economic, and institutional mechanisms mediate interactions between humans and their use of ecosystem services (Hicks and Cinner 2014). That such mechanisms collectively determine how people actually use ecosystem services has implications for how we should approach disservices. Due to the inattention by scholars to disservices, only recently has it been acknowledged that the same ecosystem function can in fact be perceived as a service or disservice depending on the social–ecological context or even be perceived simultaneously as both to the same individual (Lele et al. 2013). The few studies of disservices (e.g., Zhang et al. 2007; Dunn 2010) that do exist have documented the presence of disservices instead of focusing on possible switches between service and disservice. As a result, a conceptual framework for understanding both services and disservices remains elusive. Here, we identify when and why switching of ecosystem outputs between services and disservices is taking place.

The shifting cultivation systems of Southeast Asia—and in our study area in Laos—provide an interesting case to test switches between services and disservices because people living in these systems have both ecosystem services and disservices from the same type of species but to a varying degree across a gradient in the landscape. For example, wild animals constitute a substantial part of household food consumption and especially rodents are popular in Asia where agricultural fields provide suitable rodent habitats (Stenseth et al. 2003). At the same time, rodents are rated as the second most important constraint to cultivation with mean yield losses estimated at 20 % (Douang Boupha et al. 2010). Weeds constitute the primary constraint to cultivation in the shifting cultivation systems (Roder et al. 1995), but a large proportion of these weeds are likewise being used as food as well as medicine sources (Cruz Garcia and Price 2012). Ongoing land use transitions from subsistence to commercial agriculture are having dramatic impacts on the ecosystems, social values, and practices. The speed of these transitions differs by area, and we include villages representing various degrees of such transitions.

The purpose of this study is thus to (1) identify the availability of specific ecosystem outputs (wild animals and plants), (2) document people’s use of those animals and plants (ecosystem services), and (3) estimate the extent to which the same animals and plants cause damage to people by acting as pests and weeds (ecosystem disservices). Based on the answers to these questions, we examine the circumstances under which certain flora and fauna turn into services and disservices, and we propose revisions to existing conceptual frameworks to account for this switching between services and disservices. Our focus is on provisioning services that include a broad range of products that can be derived from forests, fallows, or agricultural fields (de Groot et al. 2010) and we restrict the analysis to animals and plants and to those taxa that occur both as ecosystem services and disservices—i.e., no attention is devoted to e.g., fungal pests or taxa that only harm crop production. We define the term pest as an animal that consumes crops during any stage of the agricultural cycle, from planting to post-harvest storage. About 12 rodent species are considered significant pests in Laos (Singleton et al. 2010) and the key pest rodent species in the upland environments is *Rattus rattus* (Brown and Khamphoukeo 2007). We define weeds as plants not purposefully cultivated and with anticipated negative effects on crop production.

## Materials and methods

### Northern Laos: A well-suited area to test ecosystem impacts on human welfare

**Fig. 1:**
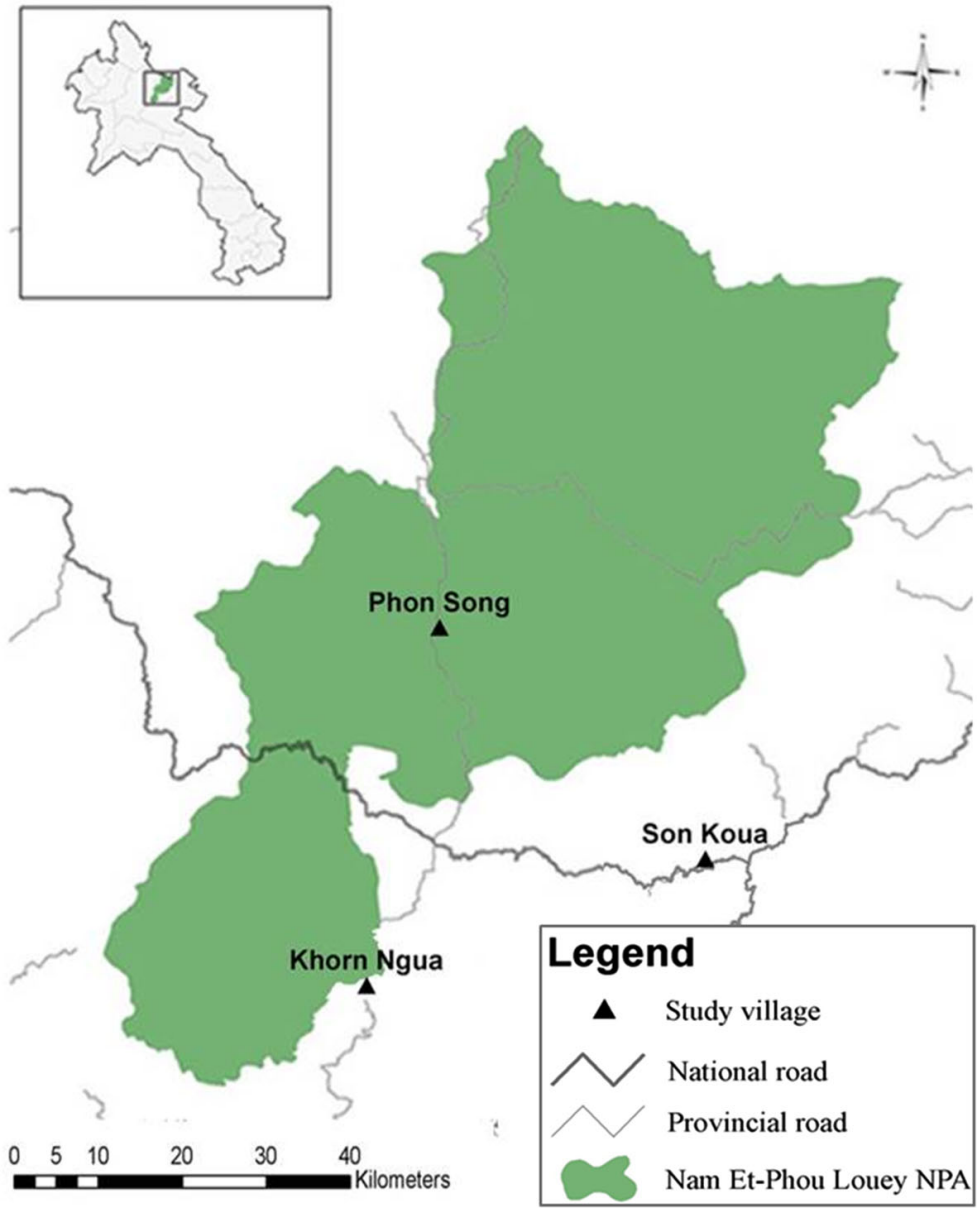
Location of the three study sites in Laos. The map also shows the Nam-Et Phou Louey National Protected Area and roads

The study took place in three villages (Khorn Ngua, Son Koua, and Phon Song), all located in northern Laos and bordering the Nam-Et Phou Louey National Protected Area (NPA) (Fig. 1). Agricultural production, primarily of rice, is the main source of sustenance for the population. Promotion of contract farming initiated by foreign investors from China and Vietnam, with a main focus on growing maize for livestock feed, has had profound impacts across the region (Messerli et al. 2009; Castella et al. 2013). Also, land use planning at the village level by the Lao Government has aimed to eliminate shifting cultivation by limiting the fallow period to 2 years maximum. Such reduced rotation times have had a strong influence on land use in northern Laos since the 1990s, though longer fallow periods do persist.

Since commercial maize was introduced in 2010 an increase in production can be seen in all three sites, but the integration of maize cultivation in the shifting cultivation systems has happened in different ways across the villages. The land use system in Khorn Ngua has changed the least and can still be described as predominantly shifting cultivation with most cultivation concentrated on steep slopes. The village of Son Koua is likewise dominated by shifting cultivation. In both villages, farmers grow upland rice or maize for 1–2 years, after which they leave the land fallow (typically 3–4 years) and shift to different plots. Maize has now been more or less integrated in the shifting cultivation system—i.e., the maize cultivation follows the shifting cultivation cycle. The agricultural season can be divided into four sub-periods: slash and burn, planting, weeding, and harvesting. No commercially produced fertilizers and pesticides are applied. Wild animals and plants are considered important sources of calories, protein, and essential vitamins. The main trapping and catching techniques for rodents are snares, single-capture traps, and pitfall traps.

In Phon Song, maize cultivation has by contrast been relatively permanent rather than integrated into shifting cultivation since its introduction. With the fallow period being omitted in these maize systems, it is, however, uncertain for how long the cultivation can be sustained without causing land degradation. Since the cultivation system no longer allows natural regeneration, the agricultural season begins with the burning rather than the slashing. Cultivation involves commercial fertilizers and pesticides and the maize is sold to external markets. Conservation policies have partially driven the inter-village difference as Phon Song is located in a core area of forest conservation. Here the establishment of strict NPA boundaries has limited access to arable land which has influenced inhabitants to accelerate agricultural intensification relative to other villages.

### Methods

To examine availability and use of different ecosystem outputs (animals and plants), four complementary methods were employed. Firstly, agricultural plots were monitored during the 2014 agricultural season from slashing in February to harvest in October in order to observe the pests and weeds present, their damage levels, and the animals and plants collected by households. The plots were established in fields belonging to 33 households (three plots per household amounting to 99 plots in total) and distributed on permanent maize fields in Phon Song (*n* = 33) and shifting cultivation rice fields in Son Koua (*n* = 33) and Khorn Ngua (*n* = 33) to highlight differences in farming systems. A stratified sample of households was used to ensure inclusion of fields at short, medium, and far away distances from the village. Secondly, collection diaries were used to estimate the amount and variety of animals and plants collected (daily records during 5 weeks, representing slash and burn, planting, weeding, harvest, and off-season for the 33 households amounting to 1155 days of collection recordings). Products derived from all landscape habitats were recorded. Thirdly, semi-structured interviews were conducted with the same 33 households (11 in each village) that participated in diary keeping and to whom the plots belonged. The aim was to validate and provide additional information on the collection of animals and plants from the field and potential problems with pests and weeds. Fourthly, participant observation was carried out to observe the 33 households’ collection of animals and plants. Villagers were accompanied when they went to collect products and on their way to the fields. These walks provided an overview of the gathering rather than the exact estimates of the extraction. For further details on the methods, see Rasmussen et al. (2016) and Appendix S1. All pests and hunted animals were identified by research assistants to taxonomic group rather than individual species level.

## Results

### Rats as a pest

We found that a broad variety of insects, diseases, and other pests affected the rice and maize production in the three villages. In total, 13 taxonomic groups were identified in the agricultural field plots, with rice stem borers, corn borers, rats, birds, and wild boar (in decreasing order of importance) causing the most damage. Six of the 13 taxonomic groups (rats, squirrels/treeshrews, wild pigs, red jungle fowl, grasshoppers, and crickets) had a dual character as they were both considered pests and collected by villagers as a food source. The interviews revealed that of those six pests with a dual character, rats were the most serious constraint to both maize and rice production.

**Fig. 2:**
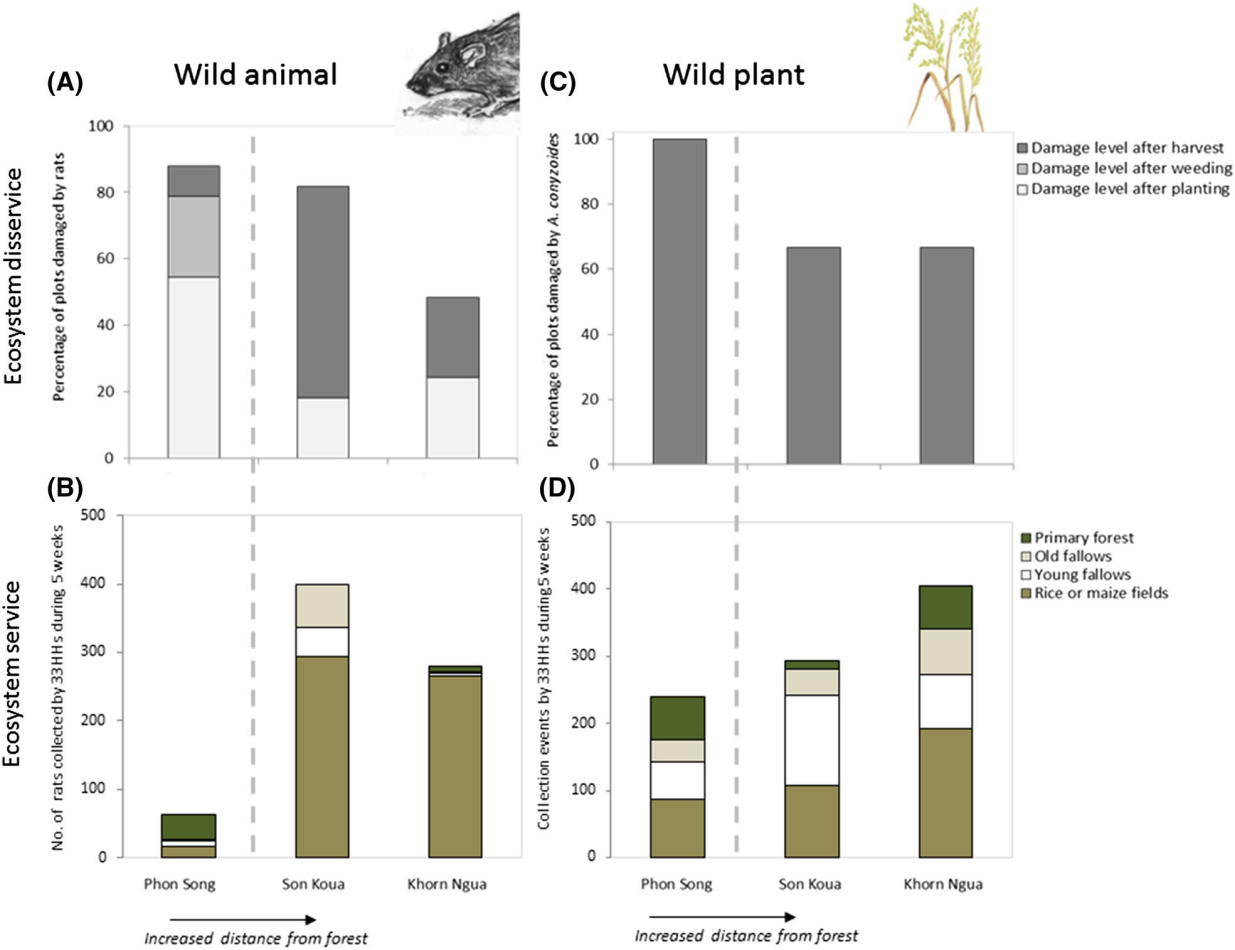
The importance of wild animals and plants as ecosystem services and disservices across three villages in northern Laos. **a** The importance of rats as a pest. Proportion of agricultural field plots affected by rats after three different growth stages (*n* = 99 plots). Plots were reported as damaged if >5 % of the area was destroyed. **b** The importance of rats as source of food. Household collection of rats for consumption (*n* = 1155 household days and 724 rats). **c** The importance of wild plants as production constraint. Proportion of agricultural field plots affected by *A. conyzoides* (*n* = 99 plots). Plots were reported as damaged if >5 % of the area was affected. **d** The importance of wild plants for consumption. Household collection of vegetables (*n* = 1155 household days and 1019 collection events). The *left side of the dashed vertical lines* represents the village with pronounced cash crop expansion located in a core area of forest, while the *right side* represents villages whose main livelihood is shifting cultivation. *HHs* households

The plot data showed that rats caused serious damage to both rice and maize at most growth stages. Across all villages, rats ate seeds and seedlings in the beginning of the growing season, but the permanent maize in Phon Song faced the highest infestation with more than half of the plots affected (Fig. 2a). After weeding, rats had caused damage in 80 % of the maize plots (*n* = 33), while they did not destroy the rice in Khorn Ngua and Son Koua during this period. Although damages increased substantially in the rice fields during the harvest period, maize continued to have the highest infestation rate (88 % of plots were affected after harvest).

Looking at the total amount of crops produced per household, we found that households faced roughly the same damage level for rice production across the villages with 8–12 % of the production being lost (Table 1). By contrast, damage levels for the maize production varied substantially across the villages. While households on average lost about 0.5 % of their maize production in Son Koua and Khorn Ngua, villagers in Phon Song reported losses in the order of 7 %. With rice prices of 0.43 US$ per kg and maize prices of 0.14 US$ per kg, the annual cost of rat damage was estimated to about 5 % of total production value in Khorn Ngua but as high as 8 % in Phon Song.

**Table 1:** Estimates of agricultural losses caused by rats and the hunting of rats as a food source in villages in and adjacent to the Nam-Et Phou Louey National Protected Area, Laos. Estimates of crop losses were obtained from household interviews (*n* = 33 households), and data on the amount of rats collected were derived from household diaries (*n* = 1155 household days and 724 rats)

	Rats as pests				Rats as a food source	
	Avg. loss/HH	Production value^a^/HH (after loss)	Loss value^a^/HH	Loss as % of total value	Avg. collection/HH/year (kg)	Collection value^b^/HH
Phon Song	~350 kg maize (7 %) ~60 kg rice (11 %)	US$ 845	~US$ 85	~8	32	~US$ 129–161
Son Koua	~10 kg maize (0.5 %) ~120 kg rice (12 %)	US$ 710	~US$ 53	~7	212	~US$ 848–1061
Khorn Ngua	~10 kg maize (0.5 %) ~100 kg rice (8 %)	US$ 796	~US$ 44	~5	130	~US$ 520–650

^a^Estimates based on a maize price of US$ 0.14 per kg and a rice price US$ 0.43 per kg

^b^Estimates based on rat prices of US$ 4–5 per kg

### Rats as a source of food

We found that rats were the most frequently hunted wild animal with 724 individuals collected for the 33 households during the 5 weeks of reporting. In Khorn Ngua and Son Koua, the hunting primarily took place in the shifting cultivation fields which accounted for 94 and 74 % of all records, respectively. By contrast, the continuously cultivated fields only contributed to 27 % of the rat collection in Phon Song (Fig. 2b).

A one-way ANOVA revealed a statistically significant difference (*F*(2179) = 6.8, *p* = 0.001) between the villages as to the number of rats collected per collection event with a fairly limited number in Phon Song (*M* = 2.2, SD = 1.7) compared to Khorn Ngua (*M* = 3.1, SD = 2.6) and Son Koua (*M* = 6.4, SD = 10.2). A post hoc Tukey test showed that Phon Song differed significantly at *p* < 0.05 from the other villages.

Households in Phon Song consumed thereby much less rat meat. While the yearly intake was about 130 and 212 kg per household in Khorn Ngua and Son Koua, respectively, it was only 32 kg in Phon Song. This finding is interesting as the highest infestation was also faced in Phon Song.

Although rat meat was rarely sold, occasional household sales were used to estimate its value. The local prices of 1 kg of rat meat ranged from US$ 4 to 5 depending on demand and supply. Based on our estimates of collected rats per household on a yearly basis, the total monetary value of rat meat would range from about US$ 1160 in Son Koua to as low as US$ 130 in Phon Song.

### Wild plants as a production constraint

Because households typically provided adequate weed control, weeds were not perceived to cause crop losses to the same degree as wild animal pests. In total, we identified 120 different weed species in the plots. In Phon Song with the permanent maize, the three most common weeds encountered were *Ageratum conyzoides*, *Triumfetta rhomboidea*, and *Clematis heracleifolia*, accounting for 20 % of all weed registrations. In all villages, households reported *A. conyzoides* as one of the most serious weeds because it is toxic to animals when consumed on a daily basis. While *A. conyzoides* was present in all plots in Phon Song, it affected less than 70 % of the plots in Son Koua and Khorn Ngua (Fig. 2c).

### Wild plants for consumption

Some weed species were appreciated by villagers. Of the 120 weed species observed, about 70 had multiple uses according to the interviewed households. Looking at the three most prevalent weeds in Son Koua and Khorn Ngua (*C. odorata*, *Conyza canadensis*, and *A. conyzoides*), we found that only *C. odorata* was collected by households. It was collected as a medicinal plant as the leaf extract was claimed to have e.g., anti-inflammatory properties. No collection of the two other species was observed. In Phon Song, none of the three most prevalent weeds were collected.

Although collection of the prevalent weeds was extremely limited, collection of other weed species took place. When households collected wild vegetables for consumption, the agricultural fields accounted for a substantial proportion. Vegetables were collected more than twice as frequently from the fields than from the old fallows and the primary forest—with similar quantity estimates per collection from the different habitats. Analogously to the observations of collected rats, we found a difference between the villages (Fig. 2d). The most frequent collection was observed in Khorn Ngua with collection of weedy vegetables from agricultural fields more than seven times per week per household, while the lowest collection frequency was found in Phon Song.

Besides being vegetable sources, many of the weeds had additional uses. For example, the bamboo species *Gigantochloa albociliata*, which was collected by more than 90 % of households in Son Koua and Khorn Ngua, could also be used as animal fodder and medicine. No collection of *G. albociliata* was observed in Phon Song.

To examine if the availability of certain weed species influenced whether or not they were actually collected, a Chi square test for independence was conducted. No significant association was found between households’ collection of five vegetables species from agricultural fields and the availability of those species. Only for the collection of *G. albociliata* a significant difference (*χ* ^2^ = 9.4, *n* = 33, *p* < 0.005) was found. Eighty-five percent of the households who had the species in their plots did also collect it indicating that this species was appreciated. For the remaining four of the five most frequently collected weed species, presence and availability of ‘beneficial’ weeds did not equate to collection and use.

Looking specifically at the use of weeds for medicinal purposes, we found that many potentially useful species went unused. The diaries revealed that only 8 of the households had collected medicinal plants from agricultural fields during the 5 weeks of reporting, totaling just 12 collection events across all households. The most frequently collected medicinal plant across all land use types (fallows, forests, and fields) was *Eleusine indica*—a weed species present in 20 % of the field plots but mainly gathered from young fallow areas and used primarily for stomach and liver problems.

## Discussion and conclusions

### Conversion factors for switching balances between services and disservices

What were the factors that determined *when* and *why* rats and plants were perceived as more beneficial than harmful and vice versa? We argue that there are three interconnected categories of livelihood factors: the institutional and governance context promoting cash crop production, the economy and market development, and the culture and identity of farmers. In addition, we identify a fourth category of spatial location (e.g., proximity of a service to the household). We propose that these interconnected sets of factors can explain situations where the balance shifts between services and disservices, but also situations where both services and disservices co-exist.

#### Institutional and governance context

Our findings showed how the institutional and governance context influenced the use of rats and plants in several important ways. Perhaps most important was the earlier mentioned government land use planning policy which has limited fallow periods to a maximum of 2 years, while at the same time promoting expansion of cash crop production. The effects of this policy were most pronounced in Phon Song due to the location in a core area of forest conservation. The shift to more permanent cultivation led to the requirement for heavy use of agricultural inputs and, according to interviewees, reduced the availability of wild food on agricultural fields. In other words, changes in rules governing agricultural practices, driven by the promotion of cash crops, have discouraged farmers from extracting potentially useful plant species. Under the more intensive farming system, wild plants are more likely to be considered weeds than they are beneficial resources.

The changing policy context in Phon Song has had a similar effect on the utility of wild animals. The shift to more permanent maize cultivation raised the profile of rats as pests and led to the application of rodenticides. Although their application was discouraged by the Lao authorities, many illegal rodenticides were still available locally as they continued to be demanded by farmers in the pursuit of profit. As rats were amplified as pests, this use of rodenticides also reduced their appeal as a food source. Our interviews revealed that villagers had heard recommendations stating that the collection of rats for food should be avoided where rodenticides were used, due to potential health effects.

#### Economy and market development

Broader changes in the local and regional economy influenced the values bestowed upon animal and plant species. First, it mattered whether the species behaved as a normal or an inferior economic good. Demand for normal goods increases as consumers become wealthier; demand for inferior goods decreases because consumers can afford more desirable alternatives (Wilkie and Godoy 2001). Some of the species that constituted provisioning services in the three villages appeared to behave like inferior economic goods, meaning that an increase in the ability to purchase alternatives led to reduced demand. In other words, the general trend towards higher cash incomes was reducing demand for some (inferior) services. In Phon Song, rice was considered the main alternative to wild food and the stronger shift to a market economy through the expanding cash crop production appeared to have reduced the demand for rat meat and plant vegetables and medicines—as evidenced by a much lower collection of these goods. Accordingly, the value of those goods as services declined rapidly, while the costs as disservices stayed the same, indicating that the balance between service and disservice have switched.

A second and related point is that the valuation of a species is sensitive to whether it is valorized purely for subsistence use or it also has a monetary exchange value. The inferiority of goods was primarily linked to local people’s perceptions of quality (e.g., plant versus western medicines) and time allocation (e.g., as people’s labor value may rise with commercial maize production, time spent gathering wild goods may be deemed a higher opportunity cost). Given that rats and plants were seldom marketed and villagers did not purchase rat meat nor wild plants to maintain their customary diet, the monetary value of e.g., rats as meat did not translate into actual expenses. By contrast, the monetary value of rats as disservices (loss of maize) was calculable—and known to farmers in Phon Song. In financial terms, rats were therefore more perceived as a disservice.

#### Culture and identity

We found that cultural factors also influenced the use of rats and plants. For example, we found limited harvest of weeds for medicinal purposes across all villages although potentially useful species were readily present in the fields. Villagers’ reasons for letting those species go unused included the construction of health centers based on Western rather than traditional medicine. Products from these centers had substituted the use of medicinal plants and this was not only a result of the changing economy but also corresponding changes in aspiration and self-identity. Our findings suggest that villagers’ lack of inclination to use medicinal plants was due to a changing cultural setting in which health centers had become a better fit with modern lifestyles and identities than the more traditional medicine practices they were replacing.

Such cultural aspects of modernization were also influencing demand for wild plants and animals for food. The modernization of agriculture in Phon Song was indeed accompanied by changing aspirations. Whereas ownership of assets such as motorbikes and tractors, according to our interviewees, rose, the cultural traditions related to wild foods seemed to be lost as agriculture became intensified and more permanent. This example illustrates how a changing cultural setting can shape a switch away from wild food collection and convert potentially useful animal and plants into disservices. But it also illustrates how economic and cultural factors are intertwined as lifestyles change with increasing market engagement.

#### Location

In addition to the three livelihood-related categories of conversion factors described above, we found that the location of ecosystem outputs also mediated the use of those outputs. A few observations substantiate this point. For example, the spatial proximity to rats and certain plants clearly influenced whether or not they turned into a service or a disservice. Whereas the forest and fallows were anticipated to account for the bulk of wild products collected, our findings showed that the majority of wild foods in the shifting cultivation systems were in fact derived from the agricultural fields—for reasons of spatial proximity to the agricultural fields, ease of collection, and abundance of desired products. Vegetables could easily be gathered while farmers were working in the fields, while the amount of time spent gathering in old fallows and primary forests was considered burdensome due to the longer distances. In Phon Song, the use of chemicals had, however, rendered the use of plants and animals from the fields undesirable. Whether the plants available in certain agricultural fields turn into a service or a disservice will thus partly depend on the spatial proximity to that field.

### A framework for the switching between services and disservices

Most existing ecosystem service frameworks are based on the implicit assumption that ecosystem outputs lead to ‘goods’ or services that provide benefits to humans. What we have illustrated above, with an empirical focus on shifting cultivation systems in Laos, is that some ecosystem outputs do not necessarily turn into goods although they have the potential to do so. Rather, they turn into disservices, they switch between being services and disservices, or they act as both services and disservices at the same time. Our findings suggest that two main categories of ecosystem outputs—animals and plants—include taxonomic groups and species that have a dual character of being both ‘good’ and ‘bad’ or a service and disservice.

**Fig. 3:**
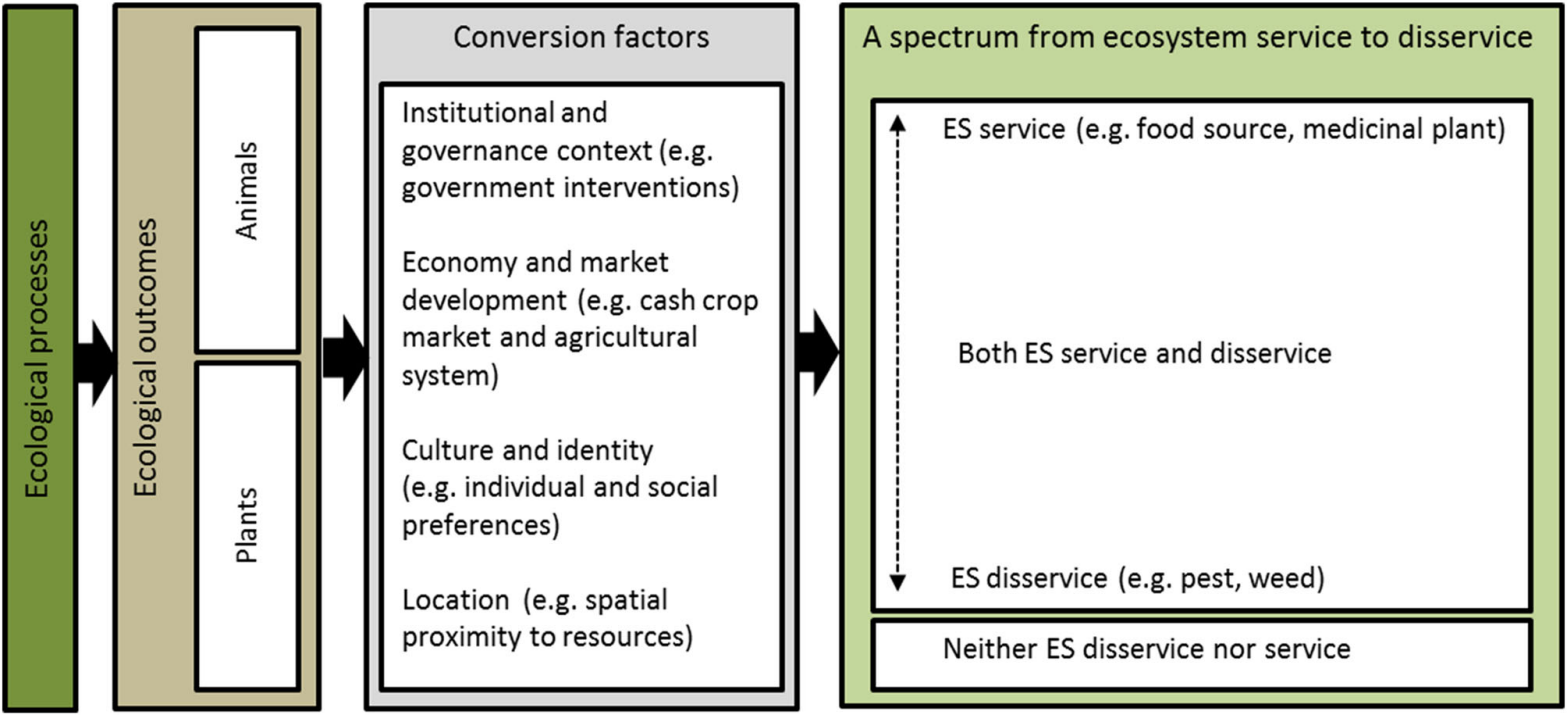
Schematic diagram that shows how ecosystem outputs in shifting cultivation systems in Laos are mediated by a range of conversion factors that determine where a certain taxon is located when, and for whom, along a spectrum from ecosystem service to ecosystem disservice. *ES* ecosystem

At the conceptual level, we propose that the switching between service and disservice is determined by what we call conversion factors—i.e., factors that mediate where certain taxonomic groups or species of animals and plants sit along a spectrum from service to disservice (Fig. 3). Based on our findings, we suggest four main categories of interlinked conversion factors: economy and market development, institutional and governance context, culture and identity, and location of ecosystem outputs. As we have outlined above, these four categories are all closely related to the agricultural system in place. We make no claim that these categories are the only conversion factors of relevance. Rather, our framework is meant to be a contribution towards a better understanding of when and why ecosystem outputs (1) turn into services rather than disservices and vice versa, (2) may act as services and disservices at the same time, and (3) are used by people in ways that influence the extent to which the same taxa cause harm or in other words act as a disservice. While the present study has focused on services and disservices in the social–ecological context of shifting cultivation systems in Laos, the suggested framework is internationally applicable given that there are many places around the world where (the same or other) plants and animals could be expected to fall along the spectrum from service to disservice (Schäckermann et al. 2015).

The proposed conversion factors build on existing theorizations of factors that determine actual use of ecosystem services. Cavender-Bares et al. (2015) argued that human values, ethics, and choices determine what is preferred and utilized by different stakeholders. Hicks and Cinner (2014) recognized that a number of ‘access mechanisms’ ultimately will increase or decrease the ecosystem services available to people. But we expand Hicks and Cinner’s categories of access mechanisms to also include spatial distances to ecosystem outputs—as we argue the distance and ease of access may determine whether outputs turn into services or disservices. Our finding that the agricultural fields provide the majority of wild food consumed also challenges the view that forest areas are the most important landscape type with regards to provisioning services (Wunder et al. 2014). Since we show how the available resources or outputs do not necessarily turn into services, the findings allow us to elaborate existing theorizations by suggesting that institutional, economic, cultural, and location factors not only mediate the ecosystem outputs’ beneficial value. Rather, the suggested factors can switch the balance between services and disservices.

The underlying argument is that presence and availability of ecosystem outputs do not necessarily mean that they will be collected and used as goods (i.e., services) (Andersson et al. 2015). If one accepts this argument at a more general level, the inadequacy of existing ecosystem service assessment framework becomes remarkably clear. When for example Mace et al. (2012) crafted their framework on linkages between biodiversity and ecosystem services, they argued that ecosystems ‘…*start with fundamental ecological and evolutionary processes and leads through final ecosystem services to the ecosystem components and outputs from which humans directly derive good and benefits*.’ Values are thereby ascribed to the ecosystem—nature becomes an active provider of services (Lele 2013). This inattention to social processes, the omission of disservices and the downplaying of possible switches between services and disservices is not just a simplifying assumption in such existing frameworks, but may potentially lead to overlooking a whole range of today’s environmental problems, from local to global (Lele 2013). Recognition of this additional feature of services and disservices as they are experienced by people has importance for the negotiation of trade-offs between different people and groups, an emerging role of ecosystem management. As we have shown with an empirical focus on shifting cultivation systems in Laos, ecosystem service frameworks need to engage with (1) the concept of disservices, (2) the conversion factors that determine where ecosystem outputs are positioned along a spectrum from service to disservice, and (3) the social processes that are implicated in the conversion factors.

In order to translate this into a better understanding of ecosystems, we, firstly, call for studies with a broad range of spatial scales (Cumming et al. 2006). It is likely that different conversion factors determine potential switches between disservices and services when one moves from the village level to the household or regional level. The general pattern derived from our analysis is that rats as an ecosystem output primarily switch into a disservice in the permanently cultivated maize systems as opposed to a service in the subsistence-oriented shifting cultivation systems. But some conversion factors, such as location of agricultural fields, may actually have caused certain households to be positioned differently in the spectrum from disservice to service. If households get time-constrained due to, for example, far away fields and they cannot devote time to set up and maintain rat traps, rats might switch towards being a disservice.

Secondly, we urge scholars to consider a range of time scales. Our study design allowed us to account for seasonal variations, but the same ecosystem output can also generate relatively more disservices in 1 year, and relatively more services in another. Taking the available plants in the agricultural fields as an example, certain species may switch into useful medicinal plants in some years (or months, weeks, or days), while the same species otherwise are considered weeds. In this regard, the balance between service and disservice may even be mediated by a particular household suffering from the specific ailment for which the plant provides treatment in a given year.

Our findings suggest that changes are required to make ecosystem service frameworks more apt and meaningful, not only for shifting cultivation systems but in all areas where diverse landscapes provide multiple outputs to their inhabitants. This is in line with recent studies illustrating that delivery of ecosystem services is insufficient as a general argument for biodiversity conservation (e.g., Kleijn et al. 2015). Our suggested framework for addressing both services and disservices should be of particular importance to scholars interested in linkages between ecosystems and human wellbeing. But it also provides new foundation for conservation and development interventions to avoid directing investments at inappropriate targets.

## Electronic supplementary material

Below is the link to the electronic supplementary material.

Supplementary material 1 (PDF 41 kb)
