# Supplementary material for: From food to pest: Conversion factors determine switches between ecosystem services and disservices
Source: Ambio. 2016 Sep 2;46(2):173–83. doi: 10.1007/s13280-016-0813-6 (PMC5274617; doi:10.1007/s13280-016-0813-6)
Supplement: Supplementary file 1 — Supplementary material 1 (PDF 41 kb) [file 13280_2016_813_MOESM1_ESM.pdf]

Ambio

Electronic Supplementary Material
This supplementary material has not been peer reviewed.

Title: **From food to pest: Conversion factors determine switches between ecosystem services and disservices**

Authors: Laura Vang Rasmussen, Andreas E. Christensen, Finn Danielsen, Neil Dawson, Adrian Martin, Ole Mertz, Thomas Sikor, Sithong Thongmanivong, Pheang Xaydongvanh

APPENDIX 1

Field data collection and analysis

Plot monitoring
For each of the 33 households, sample plots were laid out on the ground in the areas that households were going to cultivate for the coming agricultural season. This process was started in February 2014 before households began land preparation by slashing fallow vegetation. Three plots of 10m*10m were randomly distributed within the areas that the households expected to slash and cultivate, and this amounted to 99 plots for the 33 households. In Son Koua and Khorn Ngua, plots were located in the rotational rice fields, while plots were laid out in the continuous cultivated maize fields in Phon Song. Plots were demarcated marking each plot corner (SE, NE, SW, and NW) and digging a strip around each corner. Establishing permanent marked plots permitted that the plots could be revisited together with the household owning the plots during the agricultural season. When plots were laid out a qualitative description in and around the plots was recorded including a list of all plant species present in the plot and their coverage. During the establishment of plots, it was discussed with the households that they should maintain the usual cultivation practices as a changed behavior due to the plots potentially could compromise the research design.

The plots were subsequently re-visited four times during the agricultural season together with the household members cultivating the plot. These visits were done during 1) slashing, 2) planting, 3) weeding, and 4) harvesting. Each of the four visits included a detailed inventory of all the sampling plots and the following information was recorded: 1) crop-damage caused by pests monitored by animal taxonomic group and a visual estimation of the size of the area damaged; 2) dominant weed species present and a visual estimation of the coverage by each species. Each visit also involved recordings related to the households' collection of the various use-categories of animals and plants. Most of the weed species present in the agricultural fields was identified on the spot. For those species that could not be identified, they were collected, stored and brought to the Department of Biology, National University of Laos for identification.

Collection diaries
Households' collection of various animals and plants was monitored through collection diaries. Diaries were chosen as a methodology as prior studies have shown how they can be used to explore household consumption of wild vegetables with a high level of detail (Christensen 1997; Mertz et al. 2001; Lykke et al. 2002). The 33 participating households recorded the products collected, the quantity taken, the location of collection, and the final use of the product. The records were carried out on a daily basis and for a duration of one week in each of the four agricultural sub-periods (slashing, planting, weeding, and harvesting) Additionally, one week of records were done during the off-farming season. In total, the daily records amounted to 1155 days of collection recordings. Households were visited by the research assistants every evening during the recording weeks. To account for intra-household variations in collection patterns, all household members were invited to participate in the evening sessions. If some members were not available,

they were asked to tell the participating members prior to the session about their collection for that specific day.

Daily recordings were chosen over weekly in order to minimize a memory lapse. The daily visits turned out to prompt a great level of detail as the products that had been collected during the day often was shown and discussed with the research assistant. The choice of daily rather than weekly visits implied that diaries were kept in five sample weeks rather than during a whole year as this would be too costly and cumbersome for households. As the sample weeks were distributed during the year, an average collection of the various products on a yearly basis could be estimated, and the seasonality patterns in the collection could also be assessed.

Data collected through the plot monitoring and the diaries were computerized in an Access database and analyzed using SPSS version 22. Statistical analysis of data was performed using one-way ANOVA to test for differences between the three study villages.

Semi-structured interviews
When the plots were established in February 2014, semi-structured interviews were conducted with the 33 households to obtain some general information on cultivation practices, the potential problems they possibly would encounter during the agricultural season, and their general collection of various items from the fields. These interviews thereby enabled a revision of the research design which would make it more apt for capturing the most important disservices and provisioning services.

In addition, households were interviewed again after the harvest was completed. The aim was to validate the patterns emerging from the data, but also to clarify ambiguous issues found in the data. For example, reasons for using some weeds over others as food sources were discussed in detail with the households. Moreover, information on total yield losses due to pests was obtained. For all interviews, we targeted specifically the household members cultivating the fields. In more than half of the households, cultivation was done by both men and women.

It should be noted that the main focus of the study was the actual presence of weeds and pests measured through the plots and households' actual use of various animals and plants rather than households' perceptions of disservices and services.

Participant observation
In order to get a better understanding of the products households gather while they are in the fields or when walking to and back from the fields, each of the 33 households were accompanied when they went to the fields. This was done five times evenly distributed over the year, and mostly combined with plot monitoring. The field setting provided a more concrete vantage point from which the respondent could describe the problems on the fields specifically related to pests and weeds, the products collected from the fields etc. Additional details were therefore often gained through these visits, and specific and directly observable matters could more easily be discussed.

REFERENCES

Christensen H. 1997. Uses of ferns in two indigenous communities in Sarawak, Malaysia, In RJ Johns (ed.), Holttum Memorial Volume. Royal Botanic Gardens, Kew.

Lykke AM, Mertz O, Ganaba S. 2002. Food consumption in rural Burkina Faso. Ecology of Food and Nutrition 41:119-153.

Mertz O, Lykke AM, Reenberg A. 2001. Importance and seasonality of vegetable consumption and marketing in Burkina Faso. Economic Botany 55:276-289.
